# The gut–kidney axis in chronic kidney disease: mechanisms, microbial metabolites, and microbiome-targeted therapeutics

**DOI:** 10.3389/fmed.2025.1675458

**Published:** 2025-12-23

**Authors:** Sami Alobaidi

**Affiliations:** Department of Internal Medicine, University of Jeddah, Jeddah, Saudi Arabia

**Keywords:** chronic kidney disease, dysbiosis, fecal microbiota transplantation, gut microbiota, gut–kidney axis, microbiome-targeted therapy, trimethylamine N-oxide, uremic toxins

## Abstract

Chronic kidney disease (CKD) remains a major global health issue, affecting millions and presenting persistent diagnostic and therapeutic challenges. Conventional biomarkers such as serum creatinine and estimated glomerular filtration rate have well-recognized limitations, underscoring the need for novel diagnostic tools and interventions. Emerging evidence highlights the gut–kidney axis as a central contributor to CKD pathogenesis, shaped by microbial dysbiosis and altered metabolite production. Harmful metabolites such as indoxyl sulfate, p-cresyl sulfate, and trimethylamine-N-oxide promote inflammation, endothelial dysfunction, and fibrosis, while loss of protective short-chain fatty acids impairs barrier integrity and immune regulation. This review integrates mechanistic, translational, clinical, and therapeutic perspectives, offering a comprehensive and distinctive synthesis of current knowledge. We emphasize both harmful and protective microbial metabolites, incorporate the often-overlooked oral–gut–kidney axis, and highlight advances in multi-omics and computational approaches for biomarker discovery. Microbiome-targeted interventions—including dietary strategies, prebiotics, probiotics, synbiotics, oral adsorbents, and fecal microbiota transplantation—are critically evaluated with respect to efficacy, safety, and translational readiness. By bridging basic science, clinical evidence, and therapeutic implications, this review provides a forward-looking framework for integrating microbiome insights into CKD diagnosis and management. Our synthesis complements existing literature while highlighting unmet needs, thereby informing future research priorities and guiding the development of clinically relevant microbiome-based strategies.

## 1 Introduction and rationale

Chronic kidney disease (CKD) is a significant global health concern, currently affecting approximately 9.1% of the global population, corresponding to approximately 697 million individuals, with substantial contributions to morbidity, mortality, and healthcare costs (1). Early detection of CKD remains difficult due to limitations of conventional diagnostic tools. Serum creatinine and estimated glomerular filtration rate (eGFR), common indicators of kidney function, are significantly influenced by age, sex, and muscle mass, thereby reducing their accuracy, especially during CKD’s initial stages (2, 3). These limitations highlight the urgent need for novel biomarkers and advanced diagnostic methods for earlier and more accurate CKD identification and management.

Responding to these diagnostic limitations, research has increasingly explored the gut–kidney axis—a bidirectional relationship where CKD-associated uremia disrupts intestinal balance, and gut-derived microbial metabolites further contribute to kidney damage, inflammation, and fibrosis. CKD-associated dysbiosis, characterized by reduced microbial diversity and an increased prevalence of toxin-producing bacteria, enhances the generation of gut-derived uremic toxins, including indoxyl sulfate (IS), p-cresyl sulfate (PCS), and trimethylamine-N-oxide (TMAO). These metabolites exacerbate systemic inflammation, endothelial dysfunction, and renal fibrosis (4, 5).

Gut dysbiosis has been documented consistently in both pediatric and adult CKD patients. Pediatric patients with end-stage renal disease (ESRD) exhibit significant microbiota alterations independent of dialysis modality (6). In adults, dysbiotic patterns correlate closely with CKD severity and etiology, notably showing increased pro-inflammatory microbial signatures, such as an elevated abundance of Oscillibacter and Bilophila, particularly in diabetic kidney disease (7).

Given the clear connection between dysbiosis and CKD pathogenesis, microbiome-targeted therapies have gained considerable attention as potential interventions. Such therapies offer promising strategies for CKD management, although clinical outcomes vary significantly. Fecal microbiota transplantation (FMT) significantly reduced serum PCS levels and slowed CKD progression, emphasizing its therapeutic potential (8). Conversely, some probiotic interventions have produced unexpected negative results, such as increased interleukin-6 (IL-6) levels, highlighting the complexity of microbiome–host interactions (9).

Environmental pollutants further complicate the gut–kidney axis. Recent studies indicate that exposure to common pollutants, such as per- and polyfluoroalkyl substances (PFAS), exacerbates kidney dysfunction *via* microbiome disturbances, accounting for roughly half of their negative impact on kidney function (10).

Taken together, these findings underscore the central role of the gut microbiome in CKD pathogenesis and its potential as a therapeutic target. Unlike prior reviews, our study provides a balanced perspective by addressing both harmful metabolites (e.g., TMAO, IS, and PCS) and protective ones such as short-chain fatty acids (SCFAs). We integrate mechanistic insights with translational advances, highlighting multi-omics and computational approaches that bridge basic science with clinical application. Clinical evidence across diverse patient groups—including pediatric, dialysis, and diabetic populations—is systematically reviewed. We also emphasize therapeutic leverage points, critically appraising dietary, pharmacologic, and microbiota-directed strategies, and uniquely extend the scope to include the oral–gut–kidney axis, ethical considerations, and global health implications. Collectively, this approach provides a forward-looking, clinically oriented framework for microbiome-based CKD management.

## 2 Pathophysiology and mechanistic insights

### 2.1 Normal gut microbiome and renal physiology

The human gastrointestinal (GI) tract hosts trillions of microorganisms collectively known as the gut microbiome. These microorganisms are essential for nutrient metabolism, maintaining intestinal barrier integrity, and regulating immune responses. A balanced microbiome protects against pathogens through competitive exclusion and antimicrobial production (11).

Beyond intestinal health, the gut microbiome significantly impacts systemic physiology, particularly renal function, through metabolic and immunological interactions. Microbial fermentation of dietary fibers produces SCFAs such as acetate, propionate, and butyrate. SCFAs enter the bloodstream primarily through monocarboxylate transporters, exerting beneficial anti-inflammatory, antioxidative, and immunomodulatory effects on renal tissues (12, 13).

In the kidneys, SCFAs interact with G-protein-coupled receptors (GPCRs), including GPR41, GPR43, GPR109A, and Olfr78. GPR41 activation predominantly induces vasodilation and lowers blood pressure, whereas Olfr78 regulates renin secretion, fine-tuning renal blood flow and systemic blood pressure (13). Additionally, SCFAs act as histone deacetylase (HDAC) inhibitors, modulating gene expression linked to oxidative stress responses and immune regulation (12).

**Figure 1:**
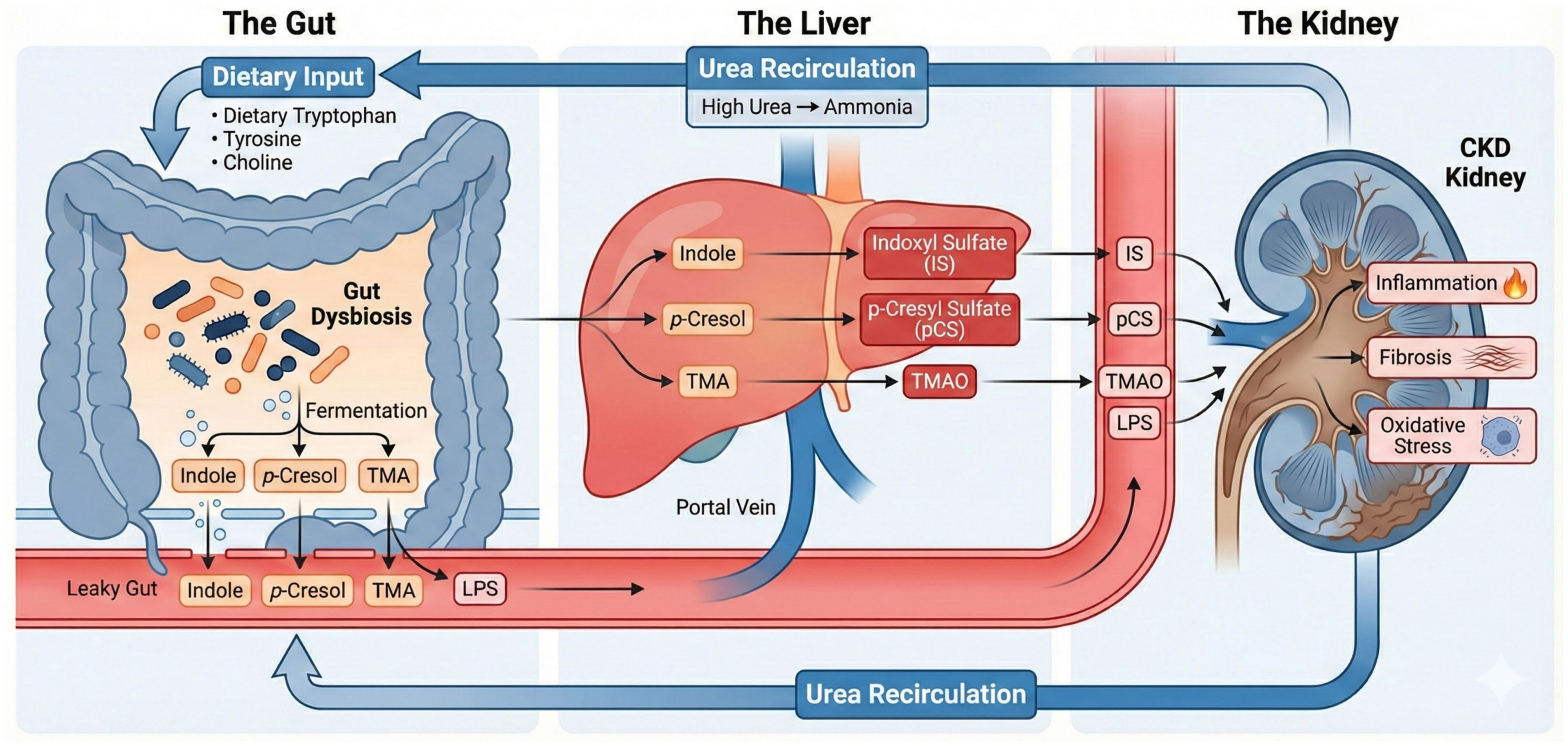
The gut–kidney axis in CKD. Dietary substrates (tryptophan, tyrosine, choline) are fermented by dysbiotic microbiota into precursors that, alongside lipopolysaccharide (LPS), translocate across the compromised “leaky gut” into the portal circulation. Hepatic metabolism converts these into the uremic toxins IS, PCS, and TMAO, which accumulate and drive renal fibrosis, inflammation, and oxidative stress. The cycle is perpetuated by urea recirculation to the gut, where hydrolysis into ammonia further promotes dysbiosis and barrier dysfunction.

The gut–kidney axis, depicted in Figure 1, illustrates the bidirectional relationship between intestinal microbiota and kidney function. In healthy individuals, gut bacteria facilitate nitrogen handling by converting urea into ammonia, reducing nitrogen waste reabsorption and enhancing renal excretion (6).

Moreover, microbial-associated molecular patterns (MAMPs) and metabolites regulate systemic immunity through interactions with intestinal epithelial and dendritic cells. These signals influence T-cell responses and renal immune tolerance, even without overt kidney injury. Maintaining microbial–immune balance is critical, as disruptions compromise gut-barrier integrity, allowing pathogen translocation and initiating systemic inflammation, as detailed further in Section 2.3.

### 2.2 Dysbiosis drivers (uremia, medications, environment)

Dysbiosis in CKD arises from complex interactions among uremia, medications, and environmental pollutants, reshaping gut microbial composition and metabolism. These alterations amplify systemic inflammation, contributing to CKD progression.

As renal function declines, urea accumulates and diffuses into the colon, where microbial urease hydrolyzes it, elevating luminal pH due to ammonia production and shifting fermentation from saccharolytic to proteolytic pathways. This shift selectively reduces beneficial SCFA-producing bacteria, such as Roseburia and Faecalibacterium, while promoting toxin-producing taxa, including Proteobacteria, Actinobacteria, Escherichia, and Clostridium. These microbial changes increase uremic toxins such as IS and PCS, directly linking dysbiosis to systemic inflammation, oxidative stress, and metabolic disturbances (14–16).

Medications used in CKD management further alter gut microbial composition. For instance, sucroferric oxyhydroxide (SFO), an iron-based phosphate binder, induces patient-specific microbiota shifts, notably enriching iron-tolerant species such as *Streptococcus salivarius*. This enrichment occurs despite relatively stable microbial diversity, driven by increased iron availability favoring bacteria less dependent on siderophore production for iron acquisition (17). Elevated serum IS and PCS were observed following SFO treatment in hemodialysis patients despite minimal overall microbiome changes, indicating functional metabolic shifts rather than broad structural alterations (18).

Environmental pollutants, particularly per- and polyfluoroalkyl substances (PFAS), significantly influence gut dysbiosis. PFAS exposure reduces beneficial SCFA-producing Lachnospiraceae and increases pro-inflammatory metabolites such as succinate and hypotaurine. These microbial alterations independently correlate with reduced eGFR and heightened systemic inflammation, intensifying gut–kidney axis disruption and CKD progression (10).

Collectively, these factors—uremic toxins, medications, and environmental pollutants—destabilize gut microbiota composition and function, impair gut-barrier integrity, and trigger systemic inflammation. These interactions create a detrimental feedback loop that perpetuates dysbiosis and accelerates renal deterioration (Figure 1).

### 2.3 Inflammatory and barrier disruption pathways

Gut dysbiosis in CKD disrupts intestinal barrier integrity, allowing microbial products such as LPS, bacterial DNA, and protein-bound uremic toxins to translocate across the compromised barrier into systemic circulation—a process termed endotoxemia. This triggers systemic inflammation and immune dysregulation, critically driving CKD progression (15, 19).

At the molecular level, gut-derived toxins activate innate immune receptors, notably Toll-like receptors (TLRs), expressed on immune and endothelial cells. Activation of TLRs, particularly TLR4 by LPS, initiates inflammatory signaling cascades, releasing pro-inflammatory cytokines including IL-6, IL-17, and tumor necrosis factor-alpha (TNF-*α*). These cytokines amplify renal fibrosis, vascular dysfunction, and systemic complications characteristic of CKD (14, 15).

Tight junction proteins, critical for maintaining gut mucosal integrity, become compromised under uremic conditions due to increased oxidative stress, inflammatory mediators, and elevated intestinal ammonia levels. Disruption of these tight junctions promotes microbial translocation and escalates cytokine production *via* NF-κB signaling pathways. Elevated serum endotoxin and oxidative stress in uremic animal models confirmed barrier dysfunction and endotoxemia as key inflammation drivers in CKD (20). Additionally, increased serum p-cresol correlated with systemic inflammation and adverse cardiovascular outcomes in CKD, further linking microbial-derived toxins to clinical complications (21).

Dysbiosis also disrupts T-cell homeostasis, causing an imbalance favoring pro-inflammatory Th17 cells over regulatory T cells (Tregs), exacerbating renal inflammation and injury. This imbalance likely results from reduced SCFA-producing bacteria, since SCFAs support Treg function and limit Th17 polarization (14).

Clinical studies have validated these immunologic mechanisms. An increased prevalence of pro-inflammatory bacterial genera, notably Escherichia, Bilophila, and Desulfovibrio, correlates with elevated inflammatory biomarkers such as C-reactive protein and cystatin C in CKD patients (16, 19). Concurrent reduction in beneficial microbes, such as Akkermansia and Faecalibacterium, further promotes chronic immune activation, barrier dysfunction, and increases susceptibility to cardiovascular and renal complications (16).

Collectively, these findings underscore the existence of a robust gut–immune–kidney axis in CKD (Figure 1). Persistent barrier dysfunction, endotoxemia, T-cell dysregulation, and microbiota-driven inflammation collectively accelerate renal decline and systemic complications. Targeting these inflammatory and barrier disruption pathways offers promising microbiota-based strategies to mitigate renal inflammation and CKD progression.

### 2.4 Key microbial metabolites in CKD progression

Gut microbial metabolites are increasingly recognized as important biomarkers and critical pathogenic factors in CKD progression (Table 1). These metabolites accumulate due to altered microbial metabolism and reduced renal clearance, exacerbating systemic inflammation, endothelial dysfunction, and direct renal injury (15).

**Table 1:** Key microbial metabolites in CKD.

Microbial metabolite	Microbial source(s)	Mechanism of pathogenesis	Clinical implications	Key references
Trimethylamine N-oxide (TMAO)	Gut bacterial metabolism of dietary choline, phosphatidylcholine, and L-carnitine	Induces systemic inflammation, oxidative stress, endothelial dysfunction, and vascular calcification	Strongly linked to increased cardiovascular risk, mortality, and potential CKD progression	Bogiatzi et al. (5) and Bao et al. (100)
Indoxyl sulfate (IS)	Gut bacterial metabolism of dietary tryptophan to indole, converted in liver to IS	Triggers inflammation, oxidative stress, endothelial dysfunction, renal fibrosis, and podocyte damage	Strong correlation with CKD progression, cardiovascular complications, mortality, and vascular calcification	Cosola et al. (64) and Arteaga-Muller et al. (8)
p-cresyl sulfate (PCS)	Colonic bacterial fermentation of dietary tyrosine and phenylalanine	Causes endothelial dysfunction, inflammation, oxidative stress, and vascular calcification	Strongly associated with CKD progression, cardiovascular morbidity, mortality, systemic inflammation	Chen et al. (7) and Bao et al. (100)
Phenylacetylglutamine	Microbial metabolism of dietary phenylalanine	Promotes systemic inflammation and elevates cardiovascular risk	Linked to increased cardiovascular morbidity in CKD patients, potentially accelerates CKD progression	Bogiatzi et al. (5)
Oxalate	Enteric degradation of dietary oxalate by *Oxalobacter formigenes* and other oxalate-degrading taxa	Increases urinary oxalate excretion, enhancing calcium oxalate crystal deposition and kidney stone formation	Strongly linked to recurrent nephrolithiasis and potential CKD progression	Ellison et al. (26)
Short-chain fatty acids (acetate, propionate, butyrate)	Fermentation of dietary fiber by SCFA-producing bacteria (e.g., Roseburia, Faecalibacterium, Bacteroidetes)	Maintain epithelial barrier integrity, fuel colonocytes, anti-inflammatory *via* GPR41/43, HDAC inhibition, AhR signaling; promote Tregs	Reduced in CKD due to depletion of SCFA-producers; linked to inflammation and CKD progression; restoration through fiber, prebiotics/probiotics, or FMT shows therapeutic potential	Arteaga-Muller et al. (8), Zhao (15), Miller et al (22), and Meijers et al. (23)

Beyond uremic toxins, SCFAs, including acetate, propionate, and butyrate, represent protective microbial metabolites that decline in CKD. Generated by bacterial fermentation of dietary fiber, SCFAs fuel colonocytes, strengthen intestinal barrier integrity, and exert anti-inflammatory effects through G-protein-coupled receptors (GPR41/43), HDAC inhibition, and aryl hydrocarbon receptor signaling. They also regulate immune balance by promoting Treg differentiation and suppressing pro-inflammatory Th17 responses. Reduced abundance of SCFA-producing taxa such as *Roseburia* and *Faecalibacterium* has been reported in CKD, correlating with increased inflammation and accelerated progression, whereas interventions including dietary fiber, probiotics, or FMT can restore SCFA production and improve renal outcomes (8, 15, 22, 23).

Among nitrogenous microbial metabolites, TMAO, IS, and PCS share common pathogenic effects, including oxidative stress, endothelial dysfunction, and inflammation, though each exerts unique mechanistic pathways (Table 1). Specifically, TMAO is associated with distinct cardiovascular implications, notably endothelial dysfunction and vascular calcification (15). IS and PCS, as protein-bound uremic toxins, exacerbate tubular injury and renal fibrosis through activation of the aryl hydrocarbon receptor (AhR) pathway, significantly impacting advanced CKD stages due to reduced renal clearance (23, 24). Recent research has highlighted microbial enzymes responsible for these metabolites as promising therapeutic targets (25).

Oxalate, a non-nitrogenous metabolite primarily degraded by *Oxalobacter formigenes*, also significantly influences CKD. Reduced abundance of *O. formigenes* increases urinary oxalate, promoting nephrolithiasis and potential CKD progression, especially in predisposed populations (26).

Collectively, these microbial metabolites play pivotal roles in CKD pathogenesis. Their microbial origins and modifiability through targeted enzymatic or metabolic interventions position them as promising therapeutic targets, as discussed further in Section 4.

### 2.5 Oral–gut–kidney axis

The oral cavity is increasingly recognized as a critical microbial interface affecting systemic health, although its contribution to CKD pathophysiology has often been overlooked. Recent evidence highlights oral dysbiosis—particularly periodontal disease—as an influential factor in CKD progression, primarily through inflammatory and immune interactions with the gut–kidney axis. Periodontal disease drives systemic inflammation by increasing microbial-induced cytokines such as IL-6 and TNF-*α*, which exacerbate gut dysbiosis and further contribute to CKD progression (14, 15, 27). Oral microbial translocation into the gut has been proposed as a mechanism that may impair intestinal barrier integrity, facilitating systemic dissemination of LPS and other microbial products, thereby sustaining the low-grade endotoxemia characteristic of CKD (14).

Importantly, the relationship between oral inflammation, gut dysbiosis, and CKD appears to be bidirectional rather than strictly sequential. On one hand, oral dysbiosis worsens CKD indirectly through systemic inflammation and gut-barrier disruption (*oral → gut → CKD*). On the other hand, inflammatory responses originating in periodontal disease may also directly accelerate CKD progression *via* systemic oxidative stress and endothelial dysfunction, independent of gut mediation (27). In turn, CKD itself can aggravate dysbiosis in both the oral and gut microbiota through uremia, acidosis, altered immune responses, and therapeutic exposures, thereby reinforcing the cycle (17, 28).

Integrating oral health strategies into CKD management may help interrupt this bidirectional cycle, complement gut-targeted therapies, and ultimately mitigate systemic inflammation and disease progression.

## 3 Clinical evidence linking microbiome and CKD outcomes

### 3.1 Microbiome composition across CKD and sub-populations

Clinical studies consistently report significant alterations in gut microbiota composition across CKD stages, characterized by reduced microbial diversity, diminished SCFA-producing taxa, and increased prevalence of toxin-producing organisms. Dysbiosis varies notably by CKD stage, dialysis method, and patient age, directly influencing clinical outcomes such as inflammation, peritonitis, and mortality (29).

#### 3.1.1 Adults with non-dialysis CKD

In adults with non-dialysis CKD, microbial alterations closely mirror the dysbiotic drivers described in Section 2. Clinical studies confirm that the expansion of *Enterobacteriaceae* and concurrent decline in SCFA-producers (e.g., *Faecalibacterium*, *Roseburia*) become increasingly pronounced in stages 3–5 (30). Crucially, these compositional changes are not merely ecological but correlate strongly with elevated circulating nephrotoxins (IS, PCS) and disease severity, providing clinical validation of the gut–kidney axis (31). Multi-omics analyses further refine these observations, showing that increases in *Enterococcus* and *Staphylococcus*—coupled with reductions in *Phascolarctobacterium*—directly correlate with declining eGFR and systemic inflammation (32). Etiology-specific signatures have also been identified; notably, membranous nephropathy patients exhibit increased opportunistic pathogens (e.g., *Klebsiella*) and decreased beneficial *Akkermansia*, suggesting the potential for tailored therapeutic strategies (33). Collectively, these findings demonstrate that early microbiota alterations in non-dialysis CKD closely mirror clinical deterioration.

#### 3.1.2 Dialysis populations

Dialysis treatments substantially reshape gut microbiota profiles. Patients undergoing colonic dialysis demonstrate enhanced beneficial SCFA-producing bacteria, such as Dialister, Phascolarctobacterium, and Bifidobacterium, compared to their non-dialysis counterparts. Colonic dialysis thus restores microbial diversity toward healthy profiles, reducing harmful Enterobacteriaceae and potentially offering therapeutic advantages (34). Unlike conventional dialysis, colonic dialysis utilizes the colonic mucosa directly to remove uremic toxins, potentially explaining its distinct microbiota-modifying effects. Conversely, ESRD patients typically experience pronounced microbiota simplification, highlighted by increased Enterococcus and reduced Faecalibacterium, correlating strongly with clinical markers like β2-microglobulin and uric acid (32). Such profound microbiota simplification in ESRD patients potentially heightens susceptibility to infectious complications and systemic inflammation. This simplification may result from prolonged dialysis treatment and dietary restrictions that significantly alter gut microbial ecology. Distinct microbiome signatures—such as elevated Lactobacillus and Yersinia, coupled with reductions in Bacteroides and Phascolarctobacterium—further associate with heightened mortality risk, underscoring microbiome profiling’s potential prognostic value in dialysis populations (29). Notably, pediatric CKD populations demonstrate distinct microbiota disruptions compared to adults, reflecting developmental and treatment-related differences.

#### 3.1.3 Pediatric CKD

Pediatric CKD populations similarly exhibit marked dysbiosis, often more pronounced than in adults. Children on peritoneal dialysis consistently show reduced microbial diversity compared to pre-dialysis or transplant patients. Specifically, increases in Enterobacteriaceae and decreases in beneficial Bifidobacteria correlate with elevated uremic toxins (IS, PCS). Additionally, oral iron supplements exacerbate dysbiosis by promoting pathogenic Proteobacteria expansion (6). These pronounced pediatric microbial shifts may partly result from immature gut microbiome establishment, unique dietary restrictions, or heightened vulnerability to treatment-related impacts. Early microbiome-targeted interventions in pediatric CKD could significantly mitigate long-term disease impacts and improve quality of life. Early and pronounced microbial disruptions in pediatric CKD underscore the urgent need for age-specific microbiome interventions to manage toxin accumulation and related clinical risks effectively.

Collectively, microbiota disruptions in CKD distinctly vary by disease stage, dialysis modality, and patient age. These variations consistently involve diminished beneficial taxa, increased toxin-producing organisms, and elevated systemic inflammation. Characterizing these microbiota differences thus forms an essential foundation for developing personalized microbiome interventions and advancing our understanding of microbiota-related clinical outcomes in CKD, discussed further in Section 3.2.

### 3.2 Association with renal function decline

Multiple clinical studies confirm that gut dysbiosis is closely associated with declining renal function in CKD. Alterations in microbiota composition, function, and metabolites correlate with reduced eGFR. Metagenomic and metabolomic analyses across CKD stages 1–5 demonstrated a progressive decrease in microbial diversity with advancing disease. Beneficial taxa such as *Prevotella* sp. 885 and *Bacteroides eggerthii* positively correlated with eGFR, whereas microbial production of nephrotoxins (particularly PCS) and genes involved in bile acid synthesis and LPS production increased in advanced CKD. Functional microbiome impairments included reduced carbohydrate-active enzymes and SCFA synthesis genes, alongside increased virulence-associated genes, significantly correlating with serum cytokines and creatinine (35).

Significant declines in beneficial genera, notably *Faecalibacterium* and *Phascolarctobacterium*, have also been reported in ESRD. These taxa negatively correlated with clinical markers (serum β2-microglobulin, blood urea nitrogen [BUN], creatinine) and positively with eGFR, whereas pathogenic genera, particularly *Enterococcus* and *Staphylococcus*, were enriched, highlighting the systemic interplay between microbiome changes and renal deterioration (32).

In autosomal dominant polycystic kidney disease (ADPKD), distinct microbiota changes have been identified, including reduced *Lactobacillus* and increased *Enterobacteriaceae*, which are closely associated with lower eGFR, underscoring the microbiome’s relevance even in hereditary CKD (36).

Analysis of NHANES data showed that higher dietary index for gut microbiota (DI-GM) scores—reflective of fiber-rich diets—correlated positively with higher eGFR and reduced CKD prevalence, particularly among women (37). Elevated circulating TMAO independently predicted CKD progression, marked by ≥40% decline in eGFR or initiation of renal replacement therapy (38).

Longitudinal evidence from a cohort of over 10,000 adults demonstrated that elevated plasma TMAO independently predicted incident CKD and accelerated eGFR decline over nearly a decade, supporting its role as a microbial biomarker and therapeutic target for CKD progression (39).

Collectively, these studies identify specific microbiota components and their metabolites as promising non-invasive biomarkers and therapeutic targets within the gut–kidney axis, reinforcing the critical role of the intestinal microbiome in CKD progression.

### 3.3 Influence on comorbidities

The systemic effects of CKD extend beyond impaired kidney filtration, encompassing various comorbidities. Growing evidence implicates gut dysbiosis and its metabolites in cardiovascular, skeletal, hematologic, and metabolic complications through interconnected inflammatory, hormonal, and immune pathways.

#### 3.3.1 Cardiovascular disease risk and gut-derived toxins

Gut microbiota-derived toxins, particularly IS, PCS, and TMAO, strongly correlate with cardiovascular complications in CKD. Uremia has been shown to disrupt intestinal epithelial tight junctions, promoting endotoxin translocation and systemic inflammation (20). IS and PCS activate the aryl hydrocarbon receptor (AhR) signaling in vascular cells, causing endothelial dysfunction and arterial stiffness (40). Toxin accumulation due to dysbiosis impairs nitric oxide signaling, thereby facilitating atherosclerosis and vascular remodeling (41).

#### 3.3.2 Mineral and bone disorders

Gut microbiota significantly influence CKD-associated mineral and bone disorders (CKD-MBD) through impacts on SCFA production and vitamin K metabolism. Dysbiosis has been shown to reduce SCFA availability, disrupting osteocalcin regulation, which is essential for bone mineralization, while dysbiosis-induced inflammation also exacerbates parathyroid hormone (PTH) dysregulation, intensifying calcium-phosphate imbalance (42). Altered SCFA profiles also contribute to vascular calcification, underscoring the microbiome’s dual impact on skeletal and vascular health (43).

#### 3.3.3 Anemia and nutritional deficiencies

The gut microbiome plays a notable role in CKD-related anemia through various pathways. IS suppresses erythropoietin (EPO) production and promotes eryptosis by interfering with AhR and hypoxia-inducible factor (HIF) pathways. Dysbiosis further compromises intestinal absorption and microbial synthesis of iron, folate, and B vitamins, essential for erythropoiesis (44). Microbiome-linked nutritional deficiencies have also been shown to complicate anemia management in CKD (42).

#### 3.3.4 Metabolic disorders

Interactions between CKD and metabolic disorders are closely linked to gut microbial imbalances. Dysbiosis-induced intestinal permeability and chronic inflammation disrupt insulin signaling, contributing to hyperglycemia (45). CKD-associated microbiota alterations promote glucose intolerance and adiposity, with SCFA-producing bacteria influencing glucagon-like peptide-1 (GLP-1) secretion and nutrient absorption (42, 46). Gut-derived metabolites, including IS, PCS, and hydrogen sulfide, act as critical mediators disrupting skeletal muscle metabolism, inducing insulin resistance, and contributing to sarcopenia, thereby defining a distinct renal–gut–muscle axis in metabolic dysfunction (47).

### 3.4 Microbiome-derived biomarkers

Advances in metagenomics and metabolomics have identified microbiome-derived signatures closely correlated with renal function and CKD progression, positioning gut microbiota as promising non-invasive biomarkers for disease monitoring, risk stratification, and patient subtyping. Metagenomic and metabolomic analyses demonstrated that CKD progression is marked by depleted microbial genes for carbohydrate-active enzymes and SCFA synthesis pathways, alongside enrichment of virulence factor genes. These microbial changes significantly correlate with serum creatinine and inflammatory cytokine levels, reflecting renal dysfunction and inflammation (35).

Biomarker profiles have been expanded by identifying specific microbial taxa, notably *Faecalibacterium* and *Phascolarctobacterium*, which negatively correlated with clinical markers such as β2-microglobulin, BUN, and creatinine, and positively with eGFR. Depletion of these beneficial genera was prominent in ESRD, suggesting their utility as biomarkers for advanced CKD. Conversely, genera such as *Enterococcus* and *Staphylococcus* were enriched in ESRD, potentially indicating adverse disease progression (32).

Stratified microbiome associations have also been observed in hereditary CKD, where distinct microbial shifts in ADPKD were characterized by decreased *Lactobacillus* and increased *Enterobacteriaceae*, closely correlating with declining eGFR, suggesting that microbiota profiling could offer subtype-specific biomarkers even in genetic forms of CKD (36).

Dietary-based biomarker insights further demonstrate that higher dietary index for gut microbiota (DI-GM) scores—indicative of fiber-rich, microbiota-friendly diets—correlated with improved kidney function and reduced CKD prevalence, particularly among women (37, 38). These dietary-responsive microbial signatures highlight their potential role in early CKD detection and personalized dietary interventions.

Although current evidence remains largely observational, the consistency of microbiome signatures across diverse populations and CKD stages supports their clinical integration. Combining microbiome-derived biomarkers with traditional renal function measures may enhance early disease detection, predict progression risk, and guide personalized CKD management (Table 2).

**Table 2:** Overview of gut microbiota composition changes in CKD.

Microbiota component	Change in CKD (↑/↓)	CKD Stage(s)/Setting	Key clinical implications	Key references
Firmicutes	↓	Advanced CKD, ESRD	Reduced SCFA production leading to increased inflammation	Gao et al. (94) Bao et al. (100)
Bacteroidetes	↑	ESRD, dialysis patients	Altered bile-acid and uremic-toxin metabolism; increased inflammatory tone	Crespo-Salgado et al. (6) and Bao et al. (100)
Actinobacteria	↓	ESRD	Loss of probiotic activity; impaired gut barrier	Crespo-Salgado et al. (6) and Gao et al. (94)
Proteobacteria	↑	ESRD, dialysis patients	Increased endotoxemia and systemic inflammation	Crespo-Salgado et al. (6) and Gao et al. (94)
Enterobacteriaceae	↑	ESRD, dialysis patients	Barrier dysfunction; systemic inflammation	Crespo-Salgado et al. (6)
Bifidobacterium	↓	ESRD, dialysis patients	Decreased SCFA and folate synthesis; weakened gut barrier	Crespo-Salgado et al. (6) and Dong et al. (77)
Faecalibacterium	↓	Advanced CKD, ESRD	Loss of anti-inflammatory capacity; barrier impairment	Gao et al. (94) and Bao et al. (100)
Ruminococcaceae	↓	Advanced CKD, ESRD	Reduced beneficial fermentation; increased toxin generation	Rossi et al. (63) and Gao et al. (94)
Lachnospiraceae	↓	Advanced CKD, ESRD	Loss of butyrate producers; barrier dysfunction	Dong et al. (77) and Gao et al. (94)
Methanobacteria	↑ in CKD 3–5 → absent in ESRD	CKD 3–5, ESRD	Altered methane metabolism; possible toxin modulation	Gao et al. (94)

## 4 Therapeutic approaches and current clinical trials

**Figure 2:**
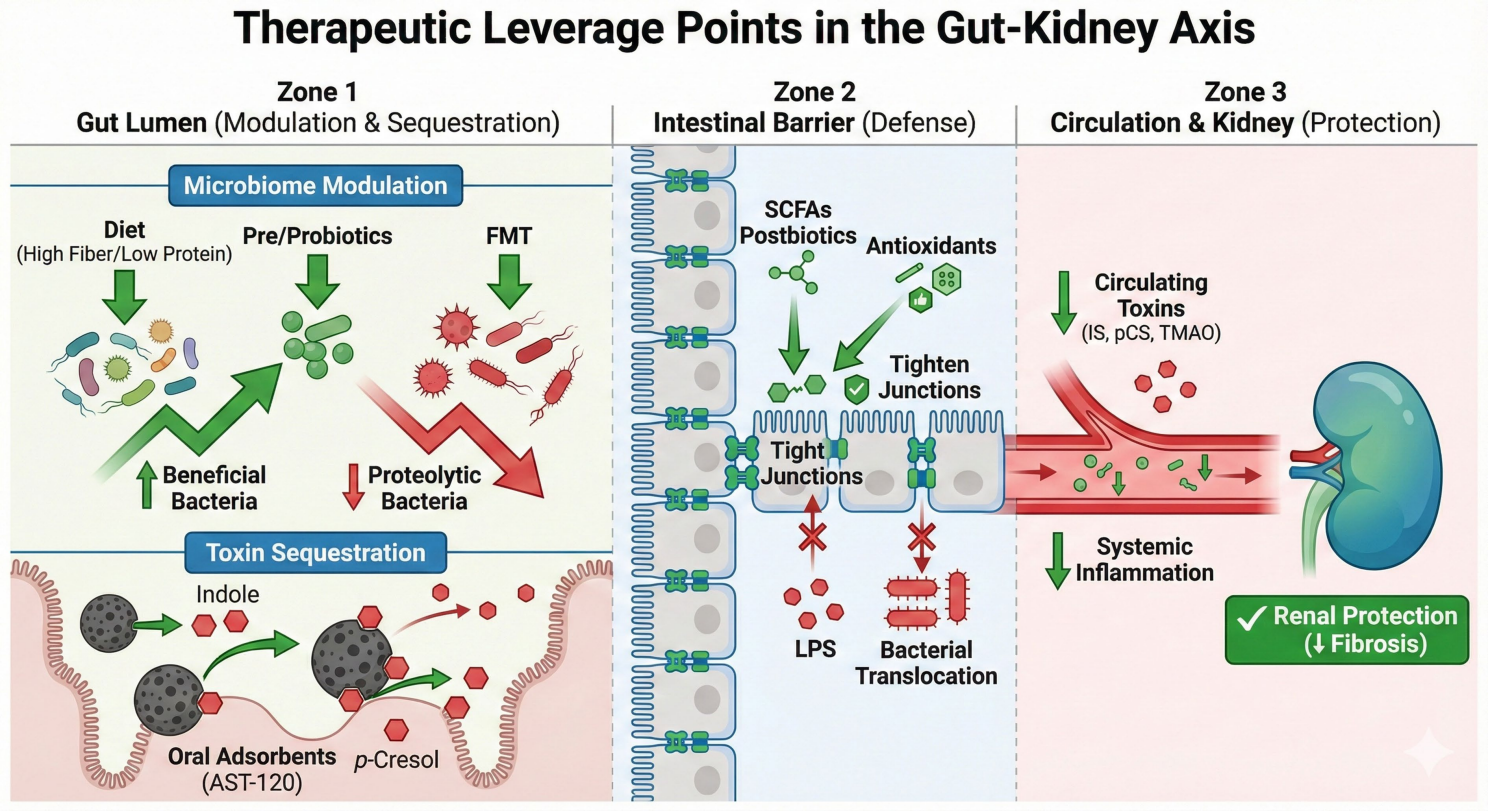
Therapeutic leverage points targeting the gut–kidney axis. Interventions are stratified by their site of action. (Zone 1: Gut Lumen) Microbiome modulation strategies (dietary fiber, prebiotics/probiotics, and FMT) restore microbial homeostasis, while oral adsorbents (e.g., AST-120) sequester toxin precursors (indole, *p*-cresol) to prevent absorption. (Zone 2: Intestinal Barrier) SCFAs, postbiotics, and antioxidants upregulate tight junction proteins, reinforcing the barrier against LPS and bacterial translocation. (Zone 3: Circulation and Kidney) Collectively, these strategies reduce the systemic burden of uremic toxins (IS, PCS, TMAO) and inflammatory mediators, thereby mitigating renal fibrosis and preserving kidney function.

Multiple strategies target discrete points along the gut–kidney axis—from dietary fiber enrichment to antioxidant therapy—aiming to correct dysbiosis, reduce toxin load, and quell inflammation (Figure 2). Various interventional studies have explored dietary modulation, prebiotics, probiotics, synbiotics, oral adsorbents, and FMT to correct CKD-associated dysbiosis, detailed in Table 3. Table 4 summarizes clinical applicability and recommendation strengths for each approach, guiding evidence-based clinical decision-making.

**Table 3:** Clinical studies evaluating microbiome-based interventions in CKD.

Reference	Intervention	Study design	Participants and CKD stage	Duration	Main findings
Schulman et al. (70)	AST-120 (uremic toxin adsorbent)	Multinational RCT	2035 patients (moderate–severe CKD)	~2 years	No benefit in CKD progression versus placebo
Rossi et al. (63)	Synbiotics (prebiotics and probiotics)	Randomized crossover trial	31 pre-dialysis CKD (eGFR 10–30)	6 weeks	Reduced PCS; increased Bifidobacterium, reduced Ruminococcaceae
Rocchetti et al. (105)	Synbiotic + DVB-PVP hemodialysis	Randomized controlled trial	11 hemodialysis patients	2 months	Significant reduction in IS and PCS
Mirzaeian et al. (106)	Synbiotic supplementation	Double-blind RCT	42 hemodialysis patients	8 weeks	No significant between-group CKD or inflammation improvements
Cosola et al. (64)	Synbiotic (probiotics, prebiotics, antioxidants)	Randomized controlled trial	23 CKD (stages 3b–4)	2 months	Reduced IS levels; improved GI symptoms and barrier function
McFarlane et al. (107) (SYNERGY II)	Resistant starch fiber + probiotics	Randomized controlled feasibility trial	68 CKD (stages 3–4)	12 months	Reduced eGFR despite beneficial microbiota changes; no significant IS/PCS reduction
Lydia et al. (104)	Synbiotic capsules (*L. acidophilus*, *B. longum*, FOS)	Randomized controlled trial	57 hemodialysis patients	2 months	Improved constipation and QoL; limited effect on uremic toxins
Zhong et al. (79)	Washed microbiota transplantation	Retrospective cohort study	53 CKD patients	Variable	Improved renal function post-treatment
Mitrović et al. (103)	Synbiotic supplementation	Double-blind RCT	34 non-dialysis CKD (eGFR 15–45)	12 weeks	Reduced IS by 21.5%; improved eGFR and inflammation markers
Sohn et al. (101)	Prebiotic (p-inulin)	Open-label pilot trial	15 CKD patients (eGFR 15–50)	12 weeks	Altered microbiota (increased Bifidobacterium, Anaerostipes); no changes in IS or PCS
Arteaga-Muller et al. (8)	FMT	Double-blind RCT	28 CKD (stages 2–4)	6 months	Reduced CKD progression (13.3% vs. placebo 53.8%); stable renal function

**Table 4:** Clinical applicability of microbiome-targeted therapies in CKD

Intervention	Typical CKD setting*	Reported benefits	Evidence quality^†^	Strength of recommendation^‡^
High-fiber/plant-based diet	CKD stages 3–5 (non-dialysis); HD/PD with serum K⁺ monitoring	Reduced IS, PCS, and IL-6; modest eGFR stability	⊕ ⊕ ⊕○ (moderate)	Conditional: First-line adjunct when serum K⁺ permits
Pre-/Pro-/Synbiotics	Stages 3–5; HD/PD	Variable reductions in IS, PCS, and modest CRP improvement	⊕ ⊕ ○○ (low)	Weak: Consider strain-specific products supported by RCT data
AST-120 oral adsorbent	Stages 3–4 (in regions where available)	Reduced IS/PCS; slower eGFR decline in select subgroups	⊕ ⊕ ⊕○ (moderate)	Conditional: Monitor gastrointestinal tolerance closely
Fecal / Washed Microbiota Transplant (FMT/WMT)	Stages 2–4 pilot studies; IgA nephropathy (IgAN)	6-mo GFR stabilization; mild adverse events	⊕ ⊕ ○○ (low)	Investigational: Restrict use to controlled clinical trials
*Setting: HD = hemodialysis; PD = peritoneal dialysis. † Evidence quality & methodology: symbols are adapted from the GRADE framework (⊕○○○ very low; ⊕ ⊕ ○○ low; ⊕ ⊕ ⊕○ moderate; ⊕ ⊕ ⊕ ⊕ high). These ratings reflect a narrative expert synthesis of the current literature reviewed in this manuscript rather than a formal systematic meta-analysis. ‡ Strength of Recommendation: ‘Conditional’ indicates that benefits may depend on specific clinical contexts (e.g., potassium levels) or patient subgroups.				

### 4.1 Dietary interventions

The gut microbiome is a promising therapeutic target in CKD, with diet being a significant modifiable factor influencing microbial composition and activity. CKD-associated dysbiosis is exacerbated by low dietary fiber and high protein intake, promoting proteolytic fermentation and uremic toxin generation. Dietary strategies emphasizing fiber enrichment and moderate protein intake may restore microbial balance and slow CKD progression.

High-fiber and plant-based diets consistently induce beneficial microbiota shifts. Very low-protein diets (VLPDs) increased beneficial taxa (Blautia, Faecalibacterium, Roseburia) and reduced pathogens (Escherichia, Klebsiella), with these effects transferable *via* FMT (48). Amylose-resistant starch supplementation in ESRD enhanced Faecalibacterium abundance and reduced systemic inflammation (49). Replacing animal proteins with plant-based sources increased Roseburia and Coprococcus, reduced PCS, and enhanced SCFA production (37). Mediterranean and Dietary Approaches to Stop Hypertension (DASH) diets enriched saccharolytic, anti-inflammatory bacteria, thereby reducing cardiovascular risk (50). A systematic review of 21 randomized controlled trials confirmed dietary fiber supplementation significantly reduced uremic toxins (PCS, IS), BUN, and inflammatory markers (IL-6, TNF-*α*), underscoring fiber’s systemic benefits (51).

Observational studies further support fiber’s clinical utility. Higher dietary fiber intake correlated with reduced serum IS, improved microbial diversity, and lower systemic inflammation in CKD patients, reinforcing its role as a dietary intervention (52).

Protein restriction strategies, including low-protein diets (LPDs) and VLPDs, also impact microbiota-derived toxins. LPDs increased Lactobacillaceae and *Streptococcus anginosus* but did not consistently reduce IS or PCS (53). Conversely, VLPDs supplemented with ketoanalogues effectively reduced uremic toxins and promoted beneficial SCFA-producing bacteria (Faecalibacterium) (31, 54). Lower protein intake correlated with reduced phenylacetylglutamine and enhanced microbial diversity (55). An umbrella review emphasized personalized LPDs, ketoanalogue-supplemented VLPDs, and tailored plant-based diets effectively reduced uremic toxins, though biomarker-guided personalization strategies remain underdeveloped (56).

Functional dietary interventions further demonstrate therapeutic potential. High-fiber granola improved bowel frequency, lowered blood pressure and serum IS, and increased beneficial taxa (Akkermansia, Bifidobacterium) in hemodialysis patients (57). Dietary metabolites related to SCFA production correlated with improved glycemic control and reduced inflammation in diabetic CKD patients (58).

Despite these documented benefits, adherence remains challenging due to complex dietary restrictions (potassium, phosphorus), limited palatability, and dietary monotony (4). Emerging solutions include microbiota-informed digital tools and personalized plant-based dietary modules to improve acceptability and compliance.

Clinical success relies on personalized dietary approaches; see Table 4 for clinical recommendations and evidence strength.

### 4.2 Prebiotic, probiotic, and synbiotic strategies

Targeting the gut microbiota through prebiotic, probiotic, and synbiotic (combined prebiotic and probiotic) supplementation represents a promising therapeutic approach in CKD. These interventions aim to restore microbial balance, reduce uremic toxins, and mitigate systemic inflammation. Clinical outcomes vary significantly due to microbial strains, dosage, duration, patient characteristics, and evaluated endpoints.

Probiotics introduce beneficial live microorganisms to modulate the gut ecosystem. Supplementation with *Lactobacillus rhamnosus* significantly reduced serum phenol and PCS in hemodialysis patients (59). Conversely, a probiotic cocktail (containing *Streptococcus thermophilus*, *Lactobacillus acidophilus*, and *Bifidobacterium longum*) administered over 3 months did not improve inflammatory markers and unexpectedly elevated serum IS and urea, highlighting strain-specific outcomes and potential probiotic risks (60).

Prebiotics, stimulating growth of beneficial native microbes, show more consistent therapeutic effects. A meta-analysis of 23 RCTs reported significant reductions in inflammatory markers (IL-6), oxidative stress, and uremic solutes, alongside expansion of SCFA-producing taxa (Faecalibacterium, Bifidobacterium), indicating more stable microbiome modulation than probiotics (61). Additionally, Gum Arabic (GA), a dietary fiber from *Acacia senegal*, consistently enriched beneficial microbiota (Bifidobacterium, Lactobacillus), enhanced SCFA (particularly butyrate) production, significantly reduced uremic toxins (IS, PCS), and modestly stabilized eGFR and inflammatory biomarkers (62).

Synbiotics, combining prebiotics and probiotics, demonstrate additive therapeutic effects. Synbiotic therapy significantly reduced serum PCS, increased Bifidobacterium abundance, and improved GI symptoms in CKD stages 4–5, though effects on IS were less pronounced (63). Similarly, synbiotics improved gut-barrier integrity, reduced free IS, and enhanced GI symptoms in CKD stages 3b–4 (64).

Meta-analyses further clarify clinical potential. A synthesis of 14 RCTs concluded that synbiotics and prebiotics consistently reduced PCS, while effects on IS varied (65). A larger meta-analysis reinforced these findings, showing probiotics and synbiotics modestly reduced inflammatory markers (CRP) and improved renal biomarkers (BUN), though effects on creatinine, eGFR, and uremic toxins remained inconsistent (33). This variability highlights the importance of intervention duration, CKD stage, and bacterial strain selection, underscoring the need for mechanistically informed trials (66).

Outcome variability emphasizes the need for precision; see Table 4 for strain-specific clinical recommendations and evidence strength.

### 4.3 Postbiotics

Postbiotics—non-viable microbial cells, cell components, or microbial metabolites—represent emerging microbiota-targeted therapies in CKD. Unlike probiotics, postbiotics exert physiological effects without requiring microbial viability, providing distinct advantages in stability, standardization, and safety, particularly for immunocompromised CKD populations.

Preclinical studies have identified promising postbiotic candidates with potential renal benefits. Lysates from *Oxalobacter formigenes* significantly reduced systemic oxalate levels, thereby preventing kidney stone formation (67). Similarly, sonicated *Lactobacillus paracasei* improved intestinal barrier integrity and reduced renal inflammation in experimental models. These beneficial effects are likely mediated by bioactive peptides, SCFAs, and the modulation of host immune signaling pathways.

Broader mechanistic insights further support postbiotics’ therapeutic potential. CKD-associated dysbiosis increases uremic toxins (IS, PCS), driven by impaired SCFA production and heightened gut permeability (68). Postbiotics may mitigate these disruptions by restoring mucosal barrier function, suppressing inflammation, and indirectly modulating microbial toxin production.

Postbiotics also integrate seamlessly into nutritional strategies targeting the gut–kidney axis, potentially stabilizing microbial ecosystems without risks associated with live bacterial supplementation (69).

Human clinical evidence for postbiotics in CKD remains limited; however, existing preclinical and mechanistic data strongly justify further exploration. The stability, favorable safety profile, and robust biological rationale of postbiotics position them as promising adjuncts or safer alternatives to probiotics, especially beneficial for vulnerable CKD patients.

Clinical evidence is preliminary; refer to Table 4 for current recommendations and evidence strength.

Future research should rigorously evaluate postbiotic formulations in clinical trials targeting gut-barrier function, toxin reduction, and host biomarkers (e.g., IL-6, TNF-*α*, CRP). Incorporating postbiotics into personalized dietary strategies or combination therapies could substantially enhance their translational potential in CKD management.

### 4.4 Pharmacologic and gene-edited microbial approaches

Pharmacologic strategies that modulate gut microbiota or reduce systemic exposure to microbial metabolites are emerging adjunctive therapies in CKD. These interventions aim to decrease uremic toxins (IS, PCS, TMAO) that are closely linked to renal and cardiovascular complications.

Carbon-based adsorbents, notably AST-120 (Kremezin®), have been extensively evaluated. Early randomized trials (EPPIC-1/EPPIC-2) did not demonstrate significant benefits in dialysis initiation or in delaying serum creatinine doubling; however, subgroup analyses indicated slower eGFR decline in selected populations (70). Recent studies further confirmed that AST-120 reduces serum IS and PCS and decreases inflammatory cytokines (e.g., TNF-*α*) (44). Mechanistically, AST-120 enhances gut-barrier integrity and promotes beneficial SCFA-producing bacteria (24).

Microbial metabolism inhibitors represent another promising intervention. TMAO, derived from dietary choline and carnitine, strongly associates with renal fibrosis and cardiovascular risk (25, 71). Dietary strategies targeting microbial TMAO synthesis effectively reduced renal injury and fibrosis in preclinical studies (72).

Gene-edited microbial approaches, involving targeted genetic modifications of microbiota to block toxin-producing pathways, offer innovative therapeutic potential. For instance, deletion of the tryptophanase gene (BT1492) in *Bacteroides thetaiotaomicron* abolished indole production, significantly reducing circulating IS in experimental models (73). These findings validate microbial genetic editing as a promising strategy for toxin reduction.

Additional microbiota-modulating compounds, such as butyrate and herbal formulations (e.g., rhubarb extracts), benefit CKD by enhancing gut-barrier integrity, reducing TMAO, and attenuating systemic inflammation (25). Such agents represent further pharmacologic options to influence microbiota activity positively.

Conventional CKD medications may unintentionally impact gut microbiota. For example, SFO alters oral and intestinal microbiota by increasing luminal iron, potentially favoring pathogenic species (17). Such observations highlight the importance of assessing microbiota effects when evaluating pharmacologic safety and efficacy in CKD management.

Clinical translation remains limited by geographic variability and experimental status; refer to Table 4 for recommendations and evidence grading. Future trials should explore synergy with dietary and microbial therapies, incorporating mechanistic biomarkers, patient stratification, and long-term renal outcomes.

### 4.5 Traditional Chinese medicine and gut microbiota in CKD

Traditional Chinese medicine (TCM) is increasingly studied in the context of the gut–kidney axis. Modern evidence indicates that TCM formulas and derived compounds can rebalance microbial composition, enhance SCFA production, reduce LPS–driven endotoxemia, and support intestinal barrier integrity. These microbiota-mediated effects are linked to improvements in systemic inflammation and renal fibrosis, positioning TCM as a promising adjunctive approach in CKD management (74, 75).

Preclinical studies provide supportive evidence. In 5/6 nephrectomy rats, the TCM formula Yishen-Qingli-Heluo Granule (YQHG) improved renal function and reduced fibrosis, though microbiota composition and barrier integrity were not assessed in this study (76). In diabetic nephropathy models, corn silk polysaccharides, a TCM-derived compound, improved creatinine and albuminuria, ameliorated histopathology, and reshaped gut taxa, including Firmicutes, Bacteroides, and Lachnospiraceae-NK4A136-group. These shifts correlated with alterations in tryptophan- and glycerophospholipid-related metabolic pathways, highlighting the microbiome as a mechanistic mediator (77).

Clinical and translational reviews reinforce these findings. Evidence shows that mild-natured, sweet-flavored TCMs and herbal polysaccharides enrich beneficial bacteria such as Bifidobacterium and Lactobacillus, while suppressing pathogenic taxa, including Enterococcus and Escherichia. These microbial changes increase SCFA levels, reduce LPS, and promote regulatory immune responses, thereby alleviating CKD-related inflammation and metabolic toxin burden. Natural products, including emodin, curcumin, and resveratrol, have also been shown to attenuate oxidative stress and renal fibrosis while modulating gut microbiota-associated pathways (74, 75). Despite these promising findings, challenges remain due to the heterogeneity of formulations, the lack of standardized dosing, and limited large-scale randomized controlled trials. Addressing these barriers will be essential to translate TCM into evidence-based, globally applicable microbiome-targeted therapies for CKD.

### 4.6 Fecal microbiota transplant

FMT offers a direct strategy to restore gut microbial homeostasis in CKD by transferring a healthy donor’s microbiota. The approach aims to reverse dysbiosis, reduce uremic toxin accumulation, and mitigate systemic inflammation. Although widely used for recurrent *Clostridioides difficile* infections, FMT is increasingly being explored as a microbiota-targeted therapy for CKD (28, 78).

Clinical evidence is emerging. The first double-blind, randomized, placebo-controlled trial of oral FMT capsules in 28 CKD patients (stages 2–4) reported a lower rate of CKD progression in the FMT group (13.3%) versus placebo (53.8%), with renal function markers remaining stable in FMT recipients. Although alpha and beta diversity metrics were unchanged, phylum-level compositional shifts were modest (↓ Firmicutes and Actinobacteria; ↑ Bacteroidetes and Proteobacteria), and at the genus level, Roseburia decreased at days 30 and 90. These mixed compositional changes suggest that any clinical signal may reflect functional/metabolic effects rather than broad taxonomic reconstitution (8). Retrospective data further indicate that washed microbiota transplantation (WMT), a modified FMT approach, improved serum creatinine, eGFR, BUN, microbiome diversity, and enhanced urinary excretion of uremic toxins (hippuric acid, indole) (79). In IgA nephropathy (IgAN), oral FMT reduced proteinuria, improved microbial diversity, and induced favorable shifts such as increases in *Prevotella copri* and *Bacteroides uniformis*. These microbial changes correlated with mucosal immune modulation, including reductions in circulating B cells, while treatment was well tolerated with minimal GI side effects (80).

Regarding safety, FMT has shown a favorable safety profile. Mild to moderate GI symptoms, including abdominal distension and altered bowel habits, were the most commonly reported adverse events, and no serious complications occurred. Rigorous donor screening, performed according to international guidelines, consistently minimized pathogen transmission risks.

Preclinical data are concordant with these clinical observations, demonstrating that transplantation of healthy donor microbiota reversed inflammation, malnutrition, and metabolic disturbances induced by uremic microbiota in animal models, highlighting FMT’s potential to restore microbial–metabolic homeostasis (78). However, significant barriers remain, including uncertain long-term safety in immunocompromised CKD patients, donor–recipient matching challenges, risks of undetected pathogens, variable microbial engraftment, and the lack of regulatory frameworks for non-infectious applications (28).

In summary, FMT represents a promising and well-tolerated microbiome-targeted strategy to slow CKD progression. Larger, multi-center trials with standardized protocols are required to confirm efficacy, refine delivery methods, and comprehensively address safety and regulatory concerns, with recommendations and evidence grading summarized in Table 4.

### 4.7 Physical activity

Physical activity (PA) is a modifiable lifestyle factor with systemic benefits extending to the gut microbiome. In CKD, where frailty, sarcopenia, and systemic inflammation are prevalent, the gut–muscle axis mediates the relationship between physical function and microbial health. Exercise positively influences gut microbial composition, fortifies intestinal-barrier integrity, and boosts SCFA generation, potentially improving renal and musculoskeletal outcomes (4, 78, 81). In murine models, aerobic exercise increased microbial diversity, enhanced tight-junction protein expression, and reduced pro-inflammatory markers such as COX-2, with these beneficial changes coinciding with increased abundance of SCFA-producing taxa, notably *Faecalibacterium prausnitzii* (82). Exercise has also been shown to preserve SCFA production and foster anti-inflammatory microbiota profiles, even under obesogenic dietary conditions (81).

Human studies align with these animal-model findings, as lower muscle mass and function in older adults were associated with reduced microbial diversity and diminished abundance of beneficial SCFA-producing taxa, including *Roseburia* and *Faecalibacterium*. Although not CKD-specific, these findings are highly relevant due to the prevalence of frailty and microbiota disruption in CKD populations (83). CKD-specific evidence demonstrates that frailty in elderly pre-dialysis CKD patients correlated with an increased abundance of pro-inflammatory genera (e.g., *Eggerthella*, *Coriobacteriaceae*, *Mogibacteriaceae*) and decreased SCFA-producing bacteria, patterns that were linked to systemic inflammation and reduced muscle mass. Given that PA enhances beneficial microbiota and modulates immune responses, it may counteract frailty-related dysbiosis in CKD (84).

Mechanistically, PA promotes gut health through multiple pathways, including enhanced SCFA production, reduced intestinal permeability, and increased expression of tight-junction proteins such as occludin and E-cadherin. Gut dysbiosis has also been shown to impair muscle adaptation to exercise, emphasizing the bidirectional importance of microbiota health in physical conditioning (85). Collectively, PA demonstrates promising microbiome-modulatory effects in CKD; however, CKD-specific interventional studies are required to determine optimal exercise regimens and confirm clinical outcomes.

## 5 Limitations and challenges

### 5.1 Heterogeneity in microbiome research

Despite rapid advancements in gut-microbiome research related to CKD, heterogeneity in study designs, methodologies, and populations significantly hampers clinical translation. Variability in sequencing methods, patient demographics, and outcome measures limits data synthesis and generalizability. Analysis of six publicly available 16S-rRNA datasets (*n* = 980) showed that the choice of variable region (e.g., V1–V2 vs. V3–V4), DNA-extraction kit, and taxonomic classifier can shift genus- and species-level assignments, complicating cross-study comparisons (86). While shotgun metagenomics yields deeper functional information, its higher analytic complexity—rather than cost alone—still limits routine clinical adoption (28).

Patient-related variability further complicates data interpretation. Marked microbial-profile differences were observed between CKD stages 3–4 and ESRD, including divergence in taxa such as *Enterococcus* and *Lachnospira* (32). However, large meta-analyses [e.g., Zhang et al. (86)] often lack detailed stratification by CKD stage, diminishing their clinical specificity (86).

Clinical trials similarly exhibit significant heterogeneity. Substantial variation was documented across 14 microbiota-targeted RCTs involving probiotic, prebiotic, and synbiotic regimens, with differences in treatment duration and outcome measures (65). Further evidence showed that rifaximin modified gut composition without improving systemic toxin or inflammatory markers, underscoring the importance of clinically meaningful endpoints (87). To advance microbiome research in CKD, establishing consensus on methodological standards is critical. Standardizing sequencing platforms, implementing clinically relevant patient stratification, and unifying outcome measures—including microbiome-specific and host-response markers—will enhance data comparability, facilitate evidence synthesis, and accelerate therapeutic development.

### 5.2 Complexity of microbiome–host interactions

Understanding the role of the gut microbiome in CKD is complicated by numerous interacting factors—including medications, diet, physical activity, and host genetics—that independently affect microbial composition and disease progression. These overlapping variables pose significant challenges for causal inference and limit the reproducibility of mechanistic insights. Medications commonly used in CKD, such as proton pump inhibitors, metformin, and antibiotics, significantly alter gut microbial diversity, potentially confounding microbiota-associated disease changes (88). Similarly, dietary restrictions typical of CKD—particularly reduced fiber and plant protein intake—suppress beneficial saccharolytic taxa and decrease SCFA production (4). Physical inactivity, prevalent in CKD, further exacerbates dysbiosis and contributes to systemic inflammation (9).

Distinguishing correlation from causation remains a persistent challenge. Bidirectional Mendelian randomization (MR) analyses of genome-wide association study (GWAS) datasets identified *Porphyromonadaceae*, *Dorea*, and *Ruminococcus torques* as CKD risk factors, while *Prevotellaceae* and *Lachnospira* were protective. Reverse MR analyses further suggested that CKD itself could alter microbial abundances, emphasizing the bidirectional complexity of host–microbiome interactions. Although MR provides a robust analytical framework, its accuracy depends heavily on instrument strength and population homogeneity, and current findings are predominantly derived from European ancestry cohorts (89).

Additionally, host genetics introduces further complexity. Genetic variability modestly yet consistently influences microbial colonization resistance, mucosal immunity, and gut-barrier function (88). Recent study has emphasized the need to address genetic confounding and pleiotropy, advocating for validation across diverse populations to adequately capture genetic and environmental heterogeneity (89). Thus, while microbiome research in CKD has identified compelling associations, definitive causal pathways remain elusive due to overlapping confounders and methodological limitations. Future advancements will require longitudinal cohort studies, integrated multi-omics approaches, and refined causal models that robustly address complex, bidirectional microbiome–host interactions across diverse populations.

### 5.3 Translation to clinical practice

Microbiota-targeted therapies hold promise for CKD management, yet their uptake in routine care is still limited owing to regulatory ambiguity, safety concerns, inter-individual variability, and implementation costs, all of which hinder broad clinical adoption. Oral FMT capsules have been shown to stabilize renal function in CKD patients but require extensive donor screening, ethical review, and specialized infrastructure, which remain unavailable in many clinical settings (8). Significant clinical benefits of standardized synbiotic formulations in reducing serum IS and intestinal permeability have also been demonstrated, but their adoption indirectly highlights the regulatory hurdles that accompany clinical implementation (64).

Safety and reproducibility pose additional challenges. No significant changes in serum indoxyl sulfate levels were observed following probiotic use in hemodialysis patients, highlighting variability in therapeutic responses (60). In some cases, probiotic supplementation even increased IL-6 levels in non-dialysis CKD patients, underscoring variability and potential safety concerns. These discrepancies emphasize the need for well-designed phase III clinical trials utilizing stratified, personalized study designs. Long-term safety data, particularly for interventions like FMT, remain limited, emphasizing the need for rigorous post-treatment safety monitoring (9).

Economic feasibility and scalability further limit adoption. Significant heterogeneity across microbiota-targeted trials complicates cost-effectiveness analyses (65). By contrast, fiber-enriched and Mediterranean dietary patterns have been shown to reduce microbiota-derived uremic toxins in patients undergoing kidney replacement therapy, offering an accessible and economically feasible therapeutic option (90). In summary, microbiome-based approaches represent a promising frontier in CKD management, but widespread implementation requires clear regulatory frameworks, personalized treatment algorithms, robust long-term safety data, and integration into existing care pathways. Future studies should prioritize harmonized trial frameworks, standardized formulations, and equitable access to microbiota-directed therapeutics.

### 5.4 Ethical considerations

As microbiome-targeted therapies gain traction in CKD, their use raises important ethical considerations surrounding informed consent, safety, and clinical justification, particularly in medically complex patients. In one FMT trial, written informed consent was obtained in line with ethical standards, underscoring the importance of adherence to these protocols (8). However, ensuring sufficient comprehension among CKD patients in routine clinical settings may pose challenges given the investigational nature and limited long-term data for microbiome therapies.

Donor safety and pathogen transmission remain significant concerns for FMT. Stringent, FDA-aligned screening protocols for viruses and multidrug-resistant organisms were implemented, and only mild GI events were reported over 6 months, but residual risks and the unknown long-term impacts of microbial engraftment remain (8). Thus, rigorous donor vetting, continuous regulatory oversight, and structured long-term surveillance are crucial ethical imperatives.

Therapeutic uncertainty further complicates ethical justification. While FMT is established for recurrent *Clostridioides difficile* infection, its application in CKD remains investigational, warranting caution due to limited efficacy and safety evidence (28). These uncertainties necessitate cautious implementation, suggesting microbiome-targeted therapies should remain primarily within controlled trials until robust safety and effectiveness data are available. In summary, microbiome-based therapies for CKD raise ethical challenges related to informed consent, donor-related risks, limited safety data, and uncertain clinical benefits. Protecting patient welfare requires confining such interventions to well-regulated trials, supported by transparent risk communication, comprehensive monitoring, and dynamic guidelines updated as new evidence emerges.

In addition to these field-level challenges, this review itself has limitations. Despite efforts to provide comprehensive coverage of the gut–kidney axis, the rapidly evolving nature of microbiome research means that some of the most recent publications may not be included. Moreover, as a narrative review, this study does not provide the quantitative rigor of a systematic meta-analysis, and its synthesis is constrained by the heterogeneity and quality of the available primary studies. These limitations should be considered when interpreting our findings, while also underscoring the importance of ongoing systematic evaluations and updated reviews as new evidence emerges.

## 6 Future directions

### 6.1 Large-scale clinical trials

Microbiota-targeted therapies demonstrate potential in CKD, yet the existing evidence base is insufficient for routine clinical implementation. Most trials enroll small patient cohorts, utilize short follow-up periods, and have highly variable designs, limiting definitive conclusions. Thus, conducting well-powered, standardized randomized controlled trials (RCTs) with clinically meaningful endpoints remains essential.

A systematic review of 14 RCTs evaluating probiotics, prebiotics, and synbiotics in CKD observed significant reductions in circulating PCS; however, effects on IS and renal outcomes were inconsistent. Marked variability in treatment duration, microbial strains, outcome measures, and study populations reduced comparability and external validity (65). Similarly, a review of 23 RCTs (n = 842) reported modest improvements in inflammatory markers, such as IL-6, and oxidative stress indicators, such as malondialdehyde, but minimal impact on renal function parameters, including eGFR and serum creatinine. These inconsistent outcomes were attributed to heterogeneity in probiotic strains, dosages, treatment durations, and patient characteristics, complicating interpretation and generalizability (61).

Collectively, these findings emphasize the need for large, multicenter trials using harmonized protocols and robust clinical endpoints. Future studies should evaluate not only uremic toxin reductions but also critical clinical outcomes, such as dialysis initiation, cardiovascular events, hospitalizations, and mortality. Ensuring diverse geographic and ethnic representation across pre-dialysis and dialysis populations will further strengthen generalizability. Ultimately, progress depends on rigorously designed RCTs capable of informing clinical guidelines and clarifying the efficacy, safety, and real-world feasibility of microbiota-based interventions.

### 6.2 Biomarkers for diagnosis and prognosis

Accurate, non-invasive biomarkers remain a critical unmet need in CKD for enabling early detection, disease stratification, and prognosis (Table 5). Traditional measures such as serum creatinine and eGFR frequently fail to detect early-stage disease or reliably predict progression. Emerging evidence suggests that gut microbiota signatures, either alone or integrated with metabolic profiles, hold promise as novel biomarkers for identifying CKD presence and severity.

**Table 5:** Biomarkers derived from microbiome profiling for CKD.

Biomarker	Associated microbial Taxa	Clinical relevance	Diagnostic/Predictive power	Key references
TMAO	Choline/L-carnitine utilizers—*Firmicutes* (e.g., *Clostridia*), *Proteobacteria*	Cardiovascular risk stratification; inverse kidney function marker	Independently predicts coronary atherosclerosis burden	Bogiatzi et al. (5) and Stubbs et al. (96)
IS	*Bacteroides*, *Clostridium*, *Fusobacterium*	CKD progression and cardiovascular event risk	Independently predicts dialysis initiation and cardiovascular events in CKD stages 3–5	Lin et al. (97)
PCS	*Enterobacteriaceae*, *Clostridium*, *Fusobacterium*, *Eubacterium*	Mortality and cardiovascular risk indicator	Free PCS independently predicts all-cause and cardiovascular mortality in CKD	Liabeuf et al. (91)
Phenylacetyl-glutamine (PAGln)	Phenylalanine-fermenting anaerobes	Cardiovascular risk prediction in CKD	Independently associated with carotid plaque burden	Bogiatzi et al. (5)
SCFAs, e.g., butyrate	*Faecalibacterium*, *Lachnospiraceae*, *Ruminococcaceae*	Gut-barrier integrity and inflammation marker	Reduced SCFA-producing bacteria correlate with advanced CKD stage and increased systemic inflammation	Gao et al. (94)
Microbial α-diversity (Shannon index)	Global microbiota metric	Dysbiosis severity and CKD risk	Reduced diversity associates with advanced CKD and prospectively predicts CKD progression	Hellman et al. (93) and Gao et al. (94)
*Oxalobacter formigenes* abundance	*Oxalobacter formigenes*	Nephrolithiasis risk marker	Absence independently associated with recurrent calcium oxalate stone formation	Kaufman et al. (102)

Integrated metagenomic and metabolomic profiling across CKD stages identified reduced abundances of *Prevotella* sp. 885 and *Bacteroides eggerthii*, alongside elevated PCS and caproic acid levels as stage-specific markers. Combined microbial–metabolite profiles achieved high diagnostic accuracy, with area under the curve (AUC) values between 0.90 and 0.95 for differentiating CKD stages (31). These findings align with clinical evidence supporting PCS as an independent predictor of cardiovascular and all-cause mortality in CKD (91). A meta-analysis of 980 fecal samples analyzed by 16S rRNA sequencing further demonstrated consistent depletion of the anti-inflammatory bacterium *Faecalibacterium prausnitzii* in CKD patients. Machine-learning models identified *F. prausnitzii* as the strongest microbial predictor distinguishing CKD from healthy controls, with an AUC of 0.74 (92). Reduced microbial diversity, previously linked to advanced CKD stages and increased systemic inflammation, further reinforces microbial profiles as promising diagnostic markers (93, 94).

Microbiome profiles have also been shown to distinguish CKD subtypes, with random forest models effectively differentiating membranous nephropathy, IgAN, minimal change disease, and ischemia-related kidney injury, achieving an overall AUC of 0.93 for discriminating CKD patients from healthy controls (95). Microbial-derived metabolites, such as TMAO and IS, further correlate with adverse clinical outcomes, including atherosclerosis and CKD progression, underscoring their potential diagnostic and prognostic value (96, 97). Collectively, these studies emphasize the emerging role of gut microbiota and associated metabolites as biomarkers for CKD diagnosis, staging, and subtype differentiation. Continued advancements in analytical techniques and refined multi-omics integration will enhance biomarker-driven risk stratification and personalized management in CKD.

### 6.3 Technological advances

Advances in multi-omics platforms and computational modeling have significantly transformed microbiome research in CKD, facilitating high-resolution profiling of microbial communities and their functional outputs. These technologies are increasingly essential for identifying diagnostic biomarkers, predictive signatures, and therapeutic targets for kidney diseases.

Integration of shotgun metagenomics and targeted metabolomics identified microbiota and metabolite signatures specific to CKD severity. Predictive models derived from combined omics data exhibited robust performance in CKD stage classification, emphasizing the clinical utility of integrated taxonomic and functional profiling (31). A global meta-analysis using 16S rRNA sequencing coupled with functional inference (PICRUSt2) revealed disruptions in oxidative stress and acid–base homeostasis pathways in CKD. Machine-learning classifiers leveraging these inferred microbial functions achieved an AUC of 0.74, underscoring their diagnostic potential (92).

AI and machine learning approaches have further enhanced diagnostic precision, with random forest classifiers effectively differentiating CKD patients from healthy controls and classifying CKD subtypes based on microbiome data. These findings highlight the effectiveness of AI-driven analytics in extracting clinically actionable patterns from complex microbial datasets (95). Collectively, these studies represent a shift from descriptive ecological profiling toward predictive, clinically relevant microbiome research. As multi-omics platforms, advanced bioinformatics, and machine learning tools become increasingly accessible, their integration will markedly improve early CKD diagnosis, individualized risk stratification, and personalized approaches to disease management.

### 6.4 Economic and health system considerations

As the global burden of CKD continues to rise, interventions must be clinically effective, scalable, affordable, and compatible with diverse healthcare systems. Microbiome-targeted nutritional interventions—including dietary modifications, probiotics, prebiotics, and synbiotics—offer promising adjuncts to conventional CKD therapy. Nutritional and microbiome-based interventions provide scalable, accessible, and cost-effective strategies for managing CKD, particularly compared to expensive pharmacological treatments. These interventions have demonstrated clinical benefits, favorable safety profiles, and minimal infrastructure requirements, enhancing their potential for integration across diverse healthcare settings (98).

Evidence from randomized trials shows that probiotics, prebiotics, and synbiotics can reduce uremic toxins, inflammation, and gut-barrier dysfunction in CKD patients while maintaining favorable safety profiles and low implementation barriers, making them particularly valuable in resource-limited settings (69). Broader reviews of microbiome interventions within the gut–kidney axis have also highlighted the importance of targeting microbiome-derived metabolites, although many have not explicitly evaluated economic implications (99). Collectively, nutritional and microbial supplementation strategies represent cost-effective and scalable adjuncts to standard CKD care. Their ease of delivery, safety, and adaptability make them well-suited for healthcare systems seeking to expand access and optimize limited resources.

### 6.5 Public health implications

Recognition of the gut–kidney axis has reframed CKD not only as a renal disorder but as intricately linked to diet, microbiota, and metabolic health. This shift underscores the public health importance of microbiome-centered strategies for CKD prevention and early-stage management. Evidence linking dietary patterns to gut dysbiosis and renal outcomes shows that Western diets rich in saturated fats and animal protein, but low in fiber, promote gut-derived uremic toxins such as IS and PCS, exacerbating inflammation and kidney injury. In contrast, plant-based, high-fiber diets foster beneficial saccharolytic bacteria, enhance SCFA production, and improve gut-barrier integrity. These findings support integrating microbiome-modulating interventions—including prebiotics, probiotics, synbiotics, and nephroprotective phytoconstituents—into dietary guidelines for early to moderate CKD, given their accessibility, affordability, and safety (69).

The systemic impacts of microbial dysbiosis, including elevated levels of TMAO, IS, and PCS, have been linked to CKD progression and cardiovascular complications, underscoring the therapeutic potential of microbiome and metabolomic profiling for personalized nutritional interventions (99). Fiber-rich diets further provide dual benefits for renal and cardiovascular protection, and personalized nutritional approaches can enhance patient outcomes in CKD while supporting broader public health initiatives focused on dietary education and targeted nutritional strategies (98).

### 6.6 Methodological priorities for next-generation studies

To effectively translate promising findings from earlier research into clinical practice, future studies should consider the following methodological priorities:

- Standardize sequencing and metadata pipelines—Establish consensus on hypervariable regions (for 16S rRNA sequencing) or shotgun metagenomics, shared quality control, and harmonized clinical metadata to enable robust meta-analysis.
- Conduct adequately powered multicenter RCTs—Enroll ≥150 participants with ≥12-month follow-up, using clinically relevant renal endpoints (e.g., eGFR decline, dialysis initiation) as primary outcomes.
- Incorporate mechanistic biomarkers—Measure SCFAs, IS and PCS kinetics, intestinal permeability assays, and inflammatory markers alongside clinical endpoints.
- Control dietary confounding—Implement unified dietary run-in periods or digital food-tracking tools to standardize fiber and protein intake before randomization.
- Ensure transparency and data sharing—Preregister analytic plans, deposit raw sequencing reads and analytical code in open repositories (e.g., NCBI-SRA, GitHub).

## 7 Conclusion

The gut–kidney axis has evolved from a theoretical concept to a central driver of CKD pathogenesis, offering a novel frontier for diagnostic and therapeutic innovation. While dysbiosis-induced uremic toxins like IS, PCS, and TMAO are now established contributors to renal progression and cardiovascular risk, the field must now pivot from descriptive ecological profiling to mechanistic intervention.

Future progress hinges on a dual approach. On the precision medicine front, integrating multi-omics and machine learning will be critical for validating microbiome-derived biomarkers and developing next-generation therapies, such as genetically engineered microbes and postbiotics. Simultaneously, the often-overlooked oral–gut–kidney axis and accessible nutritional strategies—such as fiber-rich diets—must be integrated into standard nephrology care to address the public health burden of CKD. Realizing this potential requires a concerted effort to standardize research protocols and to conduct large-scale longitudinal trials. Ultimately, targeting the microbiome represents a transformative opportunity not only to slow disease progression but also to improve the global quality of life for CKD patients.
